# Contrasting Conservation Outcomes for Ground-Dwelling and Aerial Insects in Masson Pine Plantations: Reduced Ground-Dwelling Insect Diversity but Comparable Aerial Insect Diversity to Natural Forests

**DOI:** 10.3390/insects17020158

**Published:** 2026-02-02

**Authors:** Ziming Wei, Huanhuan Liu, Chenyang Li, Xinyu Zhu, Mengli Li, Fengqun Meng

**Affiliations:** 1 Guangxi Key Laboratory of Forest Ecology and Conservation, College of Forestry, Guangxi University, Nanning 530004, China; 2 Experimental Centre of Tropical Forestry, Chinese Academy of Forestry, Pingxiang 532600, China; 3 Department of Ecology, School of Life Sciences, Nanjing University, Nanjing 210023, China; 4 Guangxi Colleges and Universities Key Laboratory for Cultivation and Utilization of Subtropical Forest Plantation, College of Forestry, Guangxi University, Nanning 530004, China

**Keywords:** Masson pine plantations, ground-dwelling insects, aerial insects, diversity, vegetation structure

## Abstract

Masson pine (*Pinus massoniana* Lamb.) is the most widely planted tree species in southern China, playing a critical role in forestry production and reforestation. Understanding the contribution of Masson pine plantations to biodiversity conservation is essential for sustainable land-use policies. We conducted comparative studies to examine the family diversity and composition of ground-dwelling and aerial insects in Masson pine plantations and adjacent natural forests at regional (spanning five forest types across Guangxi, China) and local (at Yachang, Guangxi) scales. We investigated the mechanisms driving the differences in insect community assemblages between the two forest types at the local scale. Our results indicated that aerial insect diversity and composition in Masson pine plantations were comparable to those in natural forests. However, ground-dwelling insects in plantations showed a significant decline in diversity and a notable shift in community composition, with a decrease in highly mobile omnivores (e.g., Drosophilidae and Nitidulidae) and an increase in crawling detritivores (e.g., Blattidae and Gryllidae). These patterns were consistent at both regional and local scales. At the local scale, the shift in ground-dwelling insect community composition was linked to decreased understory tree density (explaining 45.9% of the compositional variation), reduced litter Ca content (29.7%), and increased litter cover (13.5%) in plantations. To enhance ground-dwelling insect diversity in Masson pine plantations, mixed planting with broad-leaved species offers an effective management strategy. This approach both enriches litter nutrients and reduces needle litter accumulation, thereby supporting the recovery of understory vegetation.

## Simple Summary

Masson pine plantations cover huge areas of southern China and are vital for timber and reforestation. But do they support wildlife as well as natural forests? In our study, we compared insect communities in these pine plantations with those in nearby natural forests. We found that flying insects, such as flies and moths, showed similar levels of family diversity and composition in both plantation and natural forests, probably because they can easily move between habitats. In contrast, ground-dwelling insects, such as beetles and crickets, were less diverse and consisted of different taxa in the pine plantations compared to natural forests. The main reasons were fewer trees in the understory, thicker layers of pine needles, and lower Ca levels in the litter—making the habitat less suitable for many ground-dwelling insects. To help these important ground-dwelling insects thrive, forest managers can plant native broad-leaved trees together with pines. This simple change improves litter nutrition, reduces needle buildup, and allows more plants to grow underneath—creating a healthier home for insects.

## 1. Introduction

Large-scale monoculture tree plantations are established worldwide, initially to meet fuelwood and charcoal demands during early industrialization [1], later shifting toward timber production (e.g., sawlogs, pulp) [2], and recently increasingly for the provision of ecosystem services (e.g., carbon storage, soil erosion control, water provisioning) [3,4,5]. There is also growing interest in their potential to offer additional biodiversity benefits [6]. However, intense debate surrounds whether tree plantations are “green deserts” or valuable habitats for indigenous flora and fauna [7,8,9]. On the one hand, the public and many conservation scientists view monoculture plantations as “green deserts” due to their poor ecological performance. This perspective is supported by well-documented evidence of low native biodiversity across multiple taxonomic groups, including bryophytes, birds, and mammals, across a wide range of climate regions and biomes worldwide [6,9,10]. On the other hand, some argue that tree plantations can make significant contributions to biodiversity conservation and the restoration of forest species, particularly when management strategies are designed to reconcile environmental and economic objectives [8,11,12]. This perspective is supported by evidence that tree monocultures can facilitate the establishment of plant and bird communities in the understory [13,14,15] and provide essential habitat for certain endangered species [16,17]. Despite a growing body of literature on the contribution of tree plantations to forest conservation, no consensus has been reached regarding their compatibility with biodiversity conservation goals. This discrepancy in findings may primarily be attributed to two key factors. A key issue could be the use of inappropriate comparisons—most notably, the failure to benchmark plantations against the original land uses they often replace [7]. The other factor could be that conservation outcomes are likely context-dependent, varying with specific ecosystems and tree species.

China hosts the world’s largest forest plantation area [18]. Among the various planted species, Masson pine (*Pinus massoniana* Lamb.) is widely used in forestry production and reforestation in southern China due to its rapid growth, drought tolerance, and strong adaptability to infertile soils [19,20]. As a result, Masson pine has the largest planted area in China, covering 14.2 million hectares and accounting for 13.2% of the total forested land [21,22]. A better understanding of the contribution of Masson pine plantations to biodiversity conservation and other ecosystem services is essential for developing sustainable land-use policies. While previous studies have highlighted significant ecological issues in Masson pine plantations, such as soil degradation and frequent pest outbreaks [23,24], their contributions to biodiversity conservation remain inadequately quantified. Previous studies on biodiversity in Masson pine plantations have primarily focused on the responses of soil microorganisms and understory plants, and to a lesser extent soil fauna, to various management practices (e.g., logging, thinning, close-to-nature reforestation, slash burning, mixed planting, fertilization) [20,21,25,26,27,28] and different stand developmental stages [29,30]. Insects, with their high species richness, diverse ecological strategies (e.g., flying, detritivores, predators) [31], and pivotal roles in ecological processes (e.g., nutrient cycling, food webs, and pollination) [32], are valuable bioindicators of biodiversity and ecosystem functions. Additionally, their sensitivity to environmental changes also makes them essential for monitoring ecosystem health [33,34]. By assessing the contribution of Masson pine plantations to insect diversity conservation and identifying key factors that limit insect diversity, we can better guide the implementation of best management practices and promote their value for biodiversity conservation. However, to the best of our knowledge, no study has yet evaluated the role of Masson pine plantations in the conservation of insect diversity.

In this study, comparative studies were conducted to estimate insect (both ground-dwelling and aerial insects) diversity and composition in Masson pine plantations. We hypothesized a significant decline in insect diversity in Masson pine plantations compared to natural forests, based on two key augments. First, the single-layered, even-aged monoculture plantations exhibit low structural complexity and floristic diversity, which limits the availability of ecological niches, particularly in terms of food resources, shelter, and breeding sites. Second, the homogeneous pine-needle litter layer in Masson pine plantations offers limited microhabitat heterogeneity and fails to support diverse invertebrates with different ecological requirements. We further hypothesized a significant shift in insect community composition in Masson pine plantations compared to natural forests. This hypothesis is grounded in the distinct biotic and abiotic conditions created by Masson pine plantations and natural forests. Specifically, fundamental differences in floristic composition, vegetation structure, and litter properties create distinct abiotic and biotic conditions (e.g., food resources, microhabitat availability, and microclimate) that directly filter and support a different subset of insect species. Finally, we hypothesized that ground-dwelling insects would exhibit a more pronounced decline in diversity and compositional shift than aerial insects. This is supported by the inherent differences in mobility and habitat preferences between the two groups. Ground-dwelling insects, with their relatively low mobility, are more tightly linked to specific land use types. In contrast, the higher mobility of aerial insects enables them to exploit a broader range of food sources and microhabitats. Furthermore, previous studies have shown that ground-dwelling insects are more sensitive to changes in vegetation compared to aerial insects [35,36].

To test our hypotheses, we employed a multi-scale approach to compare insect diversity and composition between Masson pine plantations and the adjacent natural forests. At the regional scale, we sampled ground-dwelling insects via pitfall traps and aerial insects via Malaise traps in the two forest types at seven study sites across Guangxi, China. These sites spanned a spectrum of forest types, from northern tropical seasonal rainforest to northern subtropical evergreen broad-leaved forest. We further conducted a local-scale experiment at Yachang, Guangxi to explore the mechanisms driving the differences in insect community assemblages between Masson pine plantations and natural forests. Specifically, we quantified the family diversity and community composition of ground-dwelling and aerial insects in the two forest types, and related their compositions to environmental variables (plant, soil and litter properties). The integration of regional and local data enabled a comprehensive assessment of insect diversity and composition in Masson pine plantations and the identification of key factors governing insect assemblages.

## 2. Methods and Materials

### 2.1. Study Sites

**Figure 1:**
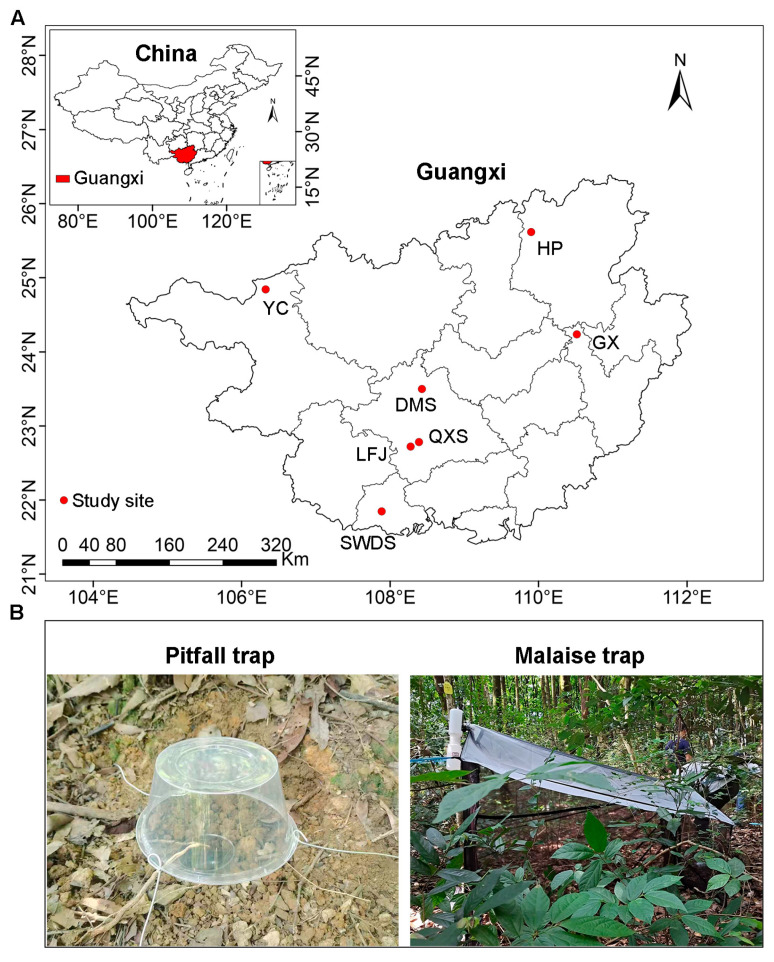
Locations of the study sites in Guangxi, China (**A**), and photographs of pitfall and Malaise traps (**B**). SWDS: Shiwandashan Nature Reserve, DMS: Damingshan Nature Reserve, HP: Huaping Nature Reserve, GX: Guxiu Nature Reserve, YC: Yachang Orchid Nature Reserve, QXS: Qingxiushan Forest Park, LFJ: Liangfengjiang Forest Park.

The study was conducted across seven sites within national nature reserves or national forest parks in Guangxi, China (Figure 1A). The region exhibits pronounced monsoonal seasonality, with mean annual precipitation decreasing westward from 2488.1 mm in the coastal southern Guangxi to 1390.3 mm in northwestern Guangxi. Concurrently, the mean annual temperature declines northward, from 23.2 °C in southern Guangxi to 16.5 °C in northern Guangxi (Table S1). This transect captures one of Asia’s steepest ecological gradients over a minimal latitudinal distance, driven by topography complexity and the Indian Ocean-East Asian monsoon interface. Consequently, our study sites encompass diverse forest types (Table S1), including northern tropical seasonal rainforest (Shiwandashan Nature Reserve, SWDS), southern subtropical evergreen broad-leaved forest (Damingshan Nature Reserve, DMS; Qingxiushan Forest Park, QXS; Liangfengjiang Forest Park, LFJ), mid-subtropical evergreen broad-leaved forest (Guxiu Nature Reserve, GX); mid-subtropical mixed evergreen and deciduous broad-leaved forest (Yachang Orchid Nature Reserve, YC), and northern subtropical evergreen broad-leaved forest (Huaping Nature Reserve, HP). All study sites are characterized by “red soils” (Ultisols). The natural forests are secondary forests that regenerated between the 1960s and 1980s, while the Masson pine plantations were 40–50 years old.

### 2.2. Insect Sampling Across Guangxi

At each study site, insect samplings were conducted in Masson plantations and the adjacent zonal natural forests from July to August 2021. Aerial insects were captured using Malaise traps, whereas ground-dwelling insects were captured using pitfall traps (Figure 1B). For aerial insects, five Malaise traps were installed at each forest type along a transect from the forest edge (~50 m from the edge) to the forest interior, with traps spaced at least 300 m apart. The Malaise traps used were of Townes type, measuring 100 cm in width, 180 cm in length, 160 cm in height at the front, and 100 cm in height at the back. They were constructed from nylon gauze, featuring a white roof and black walls, and were supplied by Guangzhou Insect Technology Service Co., Ltd. in Guangzhou, China. For ground-dwelling insects, pitfall traps were set up along the same transects as the Malaise traps. Specifically, three separate trap lines were established along each transect, with each trap line spaced at least 300 m apart. Each trap line composed of eight traps at 20 m intervals and the specimens from all eight traps in a line were pooled into a composite sample. Disposable plastic cups (7.5 cm high and 7 cm diameter) were used as containers for pitfall traps. Each trap was filled with 100–150 mL of a mixed trapping fluid (sugar:vinegar:alcohol:water = 1:2:1:20) [37]. To prevent flooding from rainfall, a plastic bowl was suspended approximately 10 cm above each cup using an iron wire support. The Malaise and pitfall traps were emptied every 10 days and a total of 3 times were collected. All specimens were then preserved in 75% ethanol for further identification. Specimens from all three collections within the same Malaise trap or pitfall trap line were pooled into a single sample. This resulted in a total of 70 samples for aerial insects and 42 samples for ground-dwelling insects across Guangxi.

All specimens were first sorted morphologically into broad categories in the laboratory and then sent to taxonomic specialists for further identification. Specifically, pitfall trap specimens were identified by specialists from Beijing Dabu Biological Science and Technology Services Co., Ltd., Beijing, China, while those belonging to Blattodea were identified by specialists from the Shanghai Entomological Museum, China. Malaise trap specimens were identified by specialists from the College of Plant Protection, China Agricultural University. The identification process, assisted by these experts, relied primarily on morphological characteristics (e.g., wings, genitalia, body structure, legs, and chelicerae) and was cross-referenced with standard taxonomic references. These included Insect Taxonomy, Insect Classification, Chinese Insects Illustrated, Insects from Mt. Shiwandashan Area of Guangxi, and Blattodea of China, along with relevant journal articles, such as those by Zahiri et al. [38,39,40,41,42,43]. In cases of taxonomic discrepancies, we adopted Insect Taxonomy as the authoritative standard for consistency. Identifications were primarily made at the family level, with a limited number proceeding to genus or species level. Consequently, all insects were designated to the family level for subsequent analyses.

### 2.3. Insect Sampling and Measurements of Forest Characteristics at Yachang, Guangxi

To unravel the mechanisms underlying insect community assemblages in Masson pine plantations and natural forests, we used a subset of the insect and environmental dataset originally collected to study the impact of tree plantation-driven fragmentation on insect diversity at Yachang, Guangxi. In this dataset, both natural forests and Masson pine plantations were set up as control plots relative to fragments. More specifically, the insect inventory was conducted from July to August 2023. In each forest type, five 20 × 20 m plots were set up, each located 500 m apart, and each plot was further divided into four 10 × 10 m quadrats. For aerial insects, a Townes-type (as previously described) Malaise trap was set up at the center of each plot, with a total of five Malaise traps set up for each forest type. For ground-dwelling insects, three quadrats within each plot were randomly selected for pitfall trap installation. In each selected quadrat, four pitfall traps (~3 m apart) were set up as previously described. Specimens were first pooled within each quadrat and then pooled within the same plot to form a composite sample per plot. The Malaise and pitfall traps were emptied every 10 days and a total of 5 times were collected. All specimens were then preserved in 75% ethanol for further identification. Specimens from all five collections within the same plot were pooled into a single sample. This resulted in a total of 10 samples for aerial insects and 10 samples for ground-dwelling insects at Yachang, Guangxi. All specimens were identified to the family level for subsequent analyses, following the identification procedures described above.

Meanwhile, we measured key forest characteristics (i.e., woody plant community, soil properties, and litter properties) that are expected to influence insect assemblages within each selected quadrat. In each selected quadrat, four evenly distributed sampling points were established to measure soil and litter properties. Woody plant communities, which provide both microhabitats and food resources for insects, were surveyed by recording all understory trees (DBH < 5 cm) and overstory trees (DBH ≥ 5 cm) within each quadrat. To assess soil properties that define microclimatic conditions for larval development, soil temperature, moisture content, and pH (in a 1:2.5 soil-deionized water suspension) were measured at each sampling point using a portable environmental monitor (LD-QX07, LEADER, Shanghai, China). Litter properties (cover, thickness, and nutrient content), which likewise serve as microhabitats and food resources for insects, were also estimated at each sampling point. For nutrient analysis, leaf litter was collected from four 50 × 50 cm^2^ areas (each centered on a sampling point) and composited into one sample per quadrat. Carbon content was measured using the combustion method with a carbon and nitrogen analyzer (Multi N/C 3100, Analytik Jena AG, Jena, Germany). Nitrogen and phosphorus contents were measured using a fully automatic flow-through analyzer (AA3, Bran Luebbe, Norderstedt, Germany) and using the molybdenum blue colorimetric method via a microplate reader (Infinite M200 PRO, TECAN, Männedorf, Switzerland), respectively, following digestion with H_2_SO_4_-H_2_O_2_. Litter K, Ca and Mg contents were measured using an atomic absorption spectrophotometer after digestion with H_2_SO_4_-H_2_O_2_. All data were standardized to a mean of 0 and variance of 1 for subsequent analysis.

### 2.4. Statistical Analysis

All statistical analyses were performed separately for each insect group (ground-dwelling and aerial insects) at the family level. We quantified family diversity (*D*) using Hill numbers of order *q* [44,45], a unified family of metrics that integrate family richness, family rarity and family dominance. We focused on four key orders: *q* = 0, *q* = 1, *q* = 2, and *q* = 3. These correspond to well-established measures: family richness index (^0^*D*), the exponential of Shannon’s index (^1^*D*), inverse Simpson’s index (^2^*D*), and inverse Berger–Parker index (^3^*D*). As the diversity order *q* increases, the metric becomes increasingly sensitive to dominant taxa and less influenced by rare ones. This continuum provides a comprehensive, multifaceted characterization of community diversity patterns. These analyses were performed using the “iNEXT” function in the R package iNEXT (version 3.0.2) [46]. For each insect group, we then evaluated whether there were significant differences in their family diversity and community composition between Masson pine plantations and natural forests, both at the regional scale across Guangxi and at the local scale in Yachang, Guangxi. Specifically, at the regional scale (i.e., across Guangxi), differences in insect diversity and community composition between forest types were assessed using generalized linear mixed models (GLMMs) and permutational multivariate analysis of variance (PERMANOVA), respectively, both incorporating study site as a random factor. At the local scale (i.e., Yachang, Guangxi), differences in insect diversity and community composition between forest types were assessed using *t*-test and PERMANOVA, respectively. The GLMM was fitted using the “lmer” function in the R package lme4 (version 1.1.38) [47], while PERMANOVA was conducted using the “adonis” function in the R package vegan (version 2.7.2) [48]. Furthermore, when a significant difference in community composition was detected, we performed a similarity percentage (SIMPER) analysis to identify the family that contributed most to the dissimilarity between forest types, using the “simper” function in the R package vegan [48]. Non-metric multidimensional scaling (NMDS) was performed to visualize the differences in insect community composition between forest types, using the “metaMDS” function in the R package vegan [48].

We further investigated the drivers (including plant, soil, and litter properties) of insect compositional differences between forest types. Due to the absence of significant compositional differences in aerial insects (see Results), this analysis was performed only for ground-dwelling insects. Additionally, the analysis was conducted separately for each plant metric (tree community composition and tree density). To do so, we evaluated how insect community dissimilarity was related to environmental dissimilarities (plant, soil, and litter properties). Community and environmental dissimilarities were quantified by Bray–Curtis and Euclidean distances, respectively, both of which were calculated with the “vegdist” function from the vegan package [48]. We then performed multiple stepwise linear regression (SMLR) to identify the best-fitting model. First, linear regression analysis was performed to examine the relationships between insect community dissimilarity and each environmental dissimilarity. Only those environmental variables that were statistically significant (i.e., *p* < 0.05) were retained for further analysis. Litter thickness, litter C content, litter N content, litter K content, and overstory tree density were excluded (Table S2). As a result, soil temperature, soil moisture content, soil pH, litter cover, litter P content, litter Ca content, litter Mg content, understory tree community composition, overstory tree community composition and understory tree density were retained for further analysis (Table S2). Next, due to strong multicollinearity among predictors, variance inflation factors (VIFs) were used to detect multicollinearity among multiple predictors, and Pearson’s correlations were employed to identify collinearity between pairs of predictors [49]. A VIF > 5 indicated that a variable was highly correlated with all other variables and was removed from further analysis. For pairs of variables with an absolute Pearson’s correlation ≥ 0.7, we retained the variable with higher predictive performance (i.e., higher *R*^2^ and lower *p* values) from the initial linear regressions. When the plant metric was measured as tree density, soil temperature, soil pH, litter P content, litter Mg content were excluded, as their VIFs were greater than 5 (ranging from 6.47 to 56.52). Soil moisture content was also excluded due to its high correlation with understory tree density (Table S3) and lower predictive power. As a result, three environmental variables were retained: litter cover, litter Ca content, and understory tree density. When the plant metric was measured as tree community composition, soil temperature, soil pH, litter P content, litter Mg content, and overstory tree community composition were excluded, as their VIFs were greater than 5 (ranging from 5.22 to 56.49). As a result, four environmental variables were retained: soil moisture content, litter cover, litter Ca content, and understory tree community composition. The retained variables were not correlated with each other (Table S3). Finally, once the final model was established, we performed multiple regressions to assess the significance of the selected explanatory variables on insect community composition. The importance of these variables and the variance explained by the models were estimated using the “lmg” function in the R package relaimpo (version 2.2.7) [50].

## 3. Results

### 3.1. Forest Characteristics

Forest characteristics were measured at the local scale in Yachang, Guangxi. For soil properties, Masson pine plantations had significantly higher soil temperature but significantly lower soil pH than natural forests (Figure S1A,B). However, there was no significant difference in soil moisture content between the two forest types (Figure S1C). For litter properties, litter cover was significantly greater in Masson pine plantations, whereas litter thickness was significantly lower (Figure S1D,E). With the exception of litter N content, which showed no significant difference, all other measured litter nutrient contents (including C, P, K, Ca, Mg) were significantly lower in Masson pine plantations than in natural forests (Figure S1F–K). For forest stand characteristics, Masson pine plantations exhibited significantly lower tree density and tree diversity both in the overstory and understory than natural forests (Figure S1L–O).

### 3.2. Characteristics of Insect Communities

At the regional scale, pitfall traps captured a total of 19,545 individuals (from 9 orders and 73 families) across Guangxi, of which 9891 (from 8 orders and 48 families) were captured in Masson pine plantations and 9654 (from 9 orders and 63 families) were captured in natural forests. At the order level, the two forest types shared similar dominant ground-dwelling insect taxa, but their relative abundances differed (Figure S2A). Blattodea (46.9 ± 10.2% for plantations and 30.9 ± 7.6% for natural forests; the same sequence for other taxa below), Orthoptera (31.6 ± 7.6% and 18.4 ± 6.8%), and Coleoptera (7.9 ± 2.0% and 20.4 ± 6.1%) were predominated in the two forest types across the seven study sites. Diptera (6.8 ± 3.2% and 21.6 ± 8.8%) was also a dominant ground-dwelling insect taxon, present at all seven study sites in natural forests and at five sites in Masson pine plantations. In contrast, Hemiptera (2.1 ± 0.4% and 4.2 ± 1.8%), Dermaptera (3.2 ± 2.5% and 2.2 ± 0.9%), and Lepidoptera (1.3 ± 0.9% and 1.3 ± 0.8%) were not dominant across the region but emerged as locally dominant taxa at a few study sites. Malaise traps captured a total of 19,323 individuals (from 12 orders and 156 families) across Guangxi, of which 10,695 (from 11 orders and 139 families) were from Masson pine plantations and 8628 (from 10 orders and 126 families) were from natural forests. At the order level, the two forest types exhibited similar aerial insect community compositions (Figure S2B). Across the seven study sites, the aerial insect communities in both forest types were dominantly composed of Diptera (39.2 ± 6.5% and 36.8 ± 5.9%), Lepidoptera (27.2 ± 5.7% and 26.3 ± 5.9%), Hemiptera (19.2 ± 3.9% and 18.3 ± 2.3%), Hymenoptera (10.3 ± 1.2% and 13.1 ± 3.2%), Coleoptera (2.5 ± 0.5% and 3.2 ± 0.8%), and Orthoptera (0.8 ± 0.4% and 1.6 ± 0.4%).

At the local scale, pitfall traps captured a total of 3023 individuals (from 8 orders and 37 families) at Yachang, Guangxi, of which 1534 (from 8 orders and 22 families) were from Masson pine plantations and 1489 (from 8 orders and 31 families) were from natural forests. At the order level, the two forest types shared similar dominant ground-dwelling insect taxa but differed in their relative abundances (Figure S3A). The ground-dwelling insect communities in both forest types were dominantly composed of Blattodea (33.1 ± 3.0% for plantations and 20.0 ± 3.7% for natural forests; the same sequence for other taxa below), Orthoptera (28.2 ± 2.7% and 12.7 ± 3.3%), Hemiptera (27.1 ± 4.5% and 20.9 ± 4.2%), Coleoptera (6.0 ± 1.1% and 10.5 ± 1.6%), and Diptera (3.2 ± 0.6% and 33.4 ± 8.1%). Malaise traps captured 13,895 individuals (from 12 orders and 149 families) at Yachang, of which 8001 (from 10 orders and 128 families) were from Masson pine plantations and 5894 (from 11 orders and 108 families) were from natural forests. At the order level, the two forest types also shared similar dominant ground-dwelling insect taxa but differed in their relative abundances (Figure S3B). The aerial insect communities in both forest types were dominantly composed of Diptera (51.4 ± 1.3% and 25.3 ± 5.3%), Hymenoptera (15.0 ± 2.0% and 7.9 ± 2.3%), Hemiptera (14.5 ± 2.3% and 36.1 ± 8.5%), Lepidoptera (10.2 ± 2.7% and 12.3 ± 4.7%), and Coleoptera (1.1 ± 0.3% and 11.2 ± 3.8%).

### 3.3. The Diversity and Community Composition of Ground-Dwelling Insects

**Figure 2:**
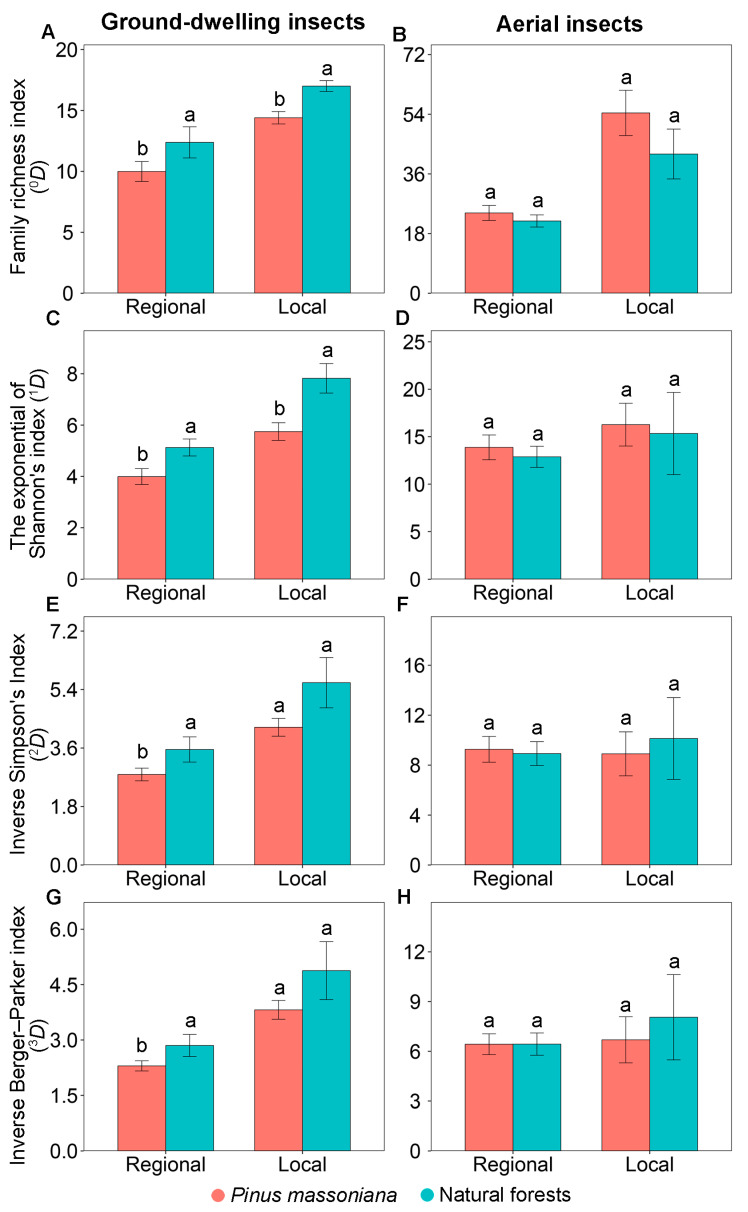
Diversity indices of ground-dwelling (**A**,**C**,**E**,**G**) and aerial (**B**,**D**,**F**,**H**) insects in *Pinus massoniana* plantations and natural forests at the regional scale across Guangxi, China and at the local scale at Yachang, Guangxi, China. Different letters at the same scale indicate significant differences between the two forest types at *p* < 0.05.

Similar patterns in the family diversity of ground-dwelling insects were observed at the regional and local scales. At both scales, family richness index (^0^*D*) and the exponential of Shannon’s index (^1^*D*) were consistently significantly lower in Masson pine plantations compared to natural forests (Figure 2A,C). However, the inverse Simpson’s index (^2^*D*) and inverse Berger–Parker index (^3^*D*) exhibited a scale-dependent pattern. While both were significantly lower in Masson pine plantations at the regional scale, no significant differences were observed at the local scale (Figure 2E,G).

**Figure 3:**
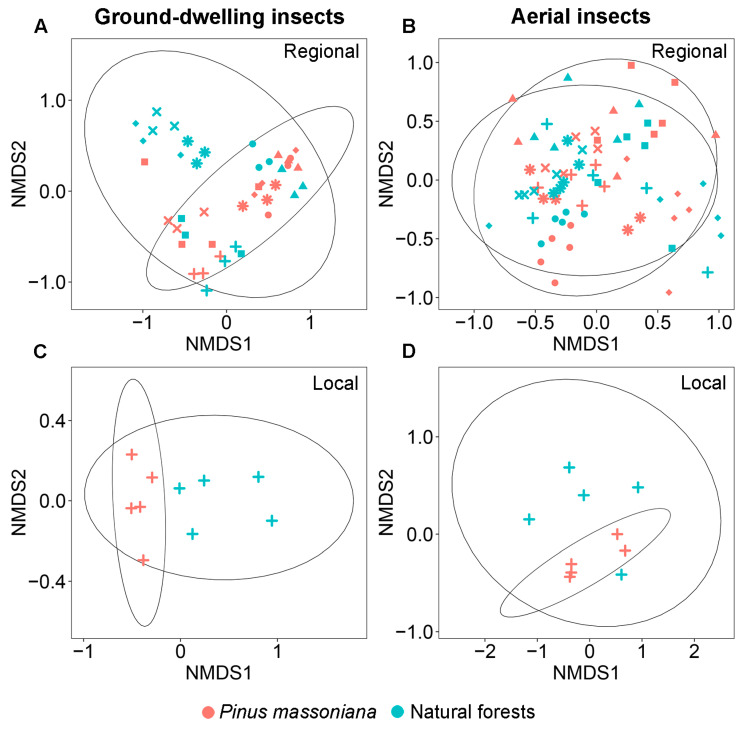
Non-metric multidimensional scaling (NMDS) of ground-dwelling (**A**,**C**) and aerial (**B**,**D**) insect community composition in *Pinus massoniana* plantations and natural forests at the regional and local scales. *: Shiwandashan Nature Reserve, ×: Damingshan Nature Reserve, ■: Huaping Nature Reserve, ◆: Guxiu Nature Reserve, +: Yachang Orchid Nature Reserve, ▲: Qingxiushan Forest Park, and ●: Liangfengjiang Forest Park.

There was a significant difference in family-level community composition of ground-dwelling insects between Masson pine plantations and natural forests at both the regional (PERMANOVA: *F* = 3.19, *p* = 0.001; Figure 3A) and local (PERMANOVA: *F* = 8.73, *p* = 0.004; Figure 3C) scales. At the regional scale, SIMPER analysis identified seven key contributors to the compositional differences between the two forest types, which together accounted for 76.1% of the observed dissimilarity (Table 1). Specifically, Blattellidae, Gryllidae, and Tetrigidae exhibited significantly higher relative abundances in plantations, whereas Drosophilidae, Nitidulidae, Scarabaeidae, and Staphylinidae were more abundant in natural forests (Table 1). At the local scale, SIMPER analysis identified six key contributors to the compositional differences between the two forest types, which together accounted for 72.0% of the observed dissimilarity. Specifically, Gryllidae, Blattidae, and Polyphagidae exhibited significantly higher relative abundances in plantations, whereas Drosophilidae, Blattellidae, and Lauxaniidae were more abundant in natural forests (Table 1). Cross-scale comparison revealed that Gryllidae (more abundant in plantations) and Drosophilidae (more abundant in natural forests) were consistent drivers of dissimilarity at both scales, contributing 34.7% and 44.1% of the observed dissimilarity at the regional and local scales, respectively (Table 1). Notably, although Blattellidae was also a key contributor to the observed dissimilarity at both scales, it exhibited a reverse trend. It was more abundant in plantations at the regional scale but more abundant in natural forests at the local scale.

Using local-scale data, we further explored the mechanisms driving differences in the community composition of ground-dwelling insects between forest types. When the plant metric was measured as tree community composition, and after controlling the multicollinearity, four environmental variables were selected (i.e., soil moisture content, litter cover, litter Ca content, and understory tree community composition). Multiple regression analysis based on these four variables revealed that soil moisture content, litter cover and litter Ca content were identified as the best predictors of the compositional variation (Table S4). However, understory tree community composition had no significant effect on the ground-dwelling insect community composition (Table S4). Together, these three variables explained approximately 74.9% of the observed compositional variation. Specifically, litter Ca content and soil moisture content were the primary contributors, accounting for 32.2% and 29.4% of the variation, respectively, while litter cover explained 13.3%. When the plant metric was measured as tree density, and after controlling the multicollinearity, three environmental variables were selected (i.e., litter cover, litter Ca content, and understory tree density). Multiple regression analysis based on these three variables revealed that understory tree density, litter cover and litter Ca content all contributed significantly to the observed compositional variation (Table 2). These three explanatory variables together explained approximately 89.1% of the compositional variation. Among them, understory tree density was the most important predictor, explaining 45.9% of the variation on its own, followed by litter Ca content (29.7%) and litter cover (13.5%), respectively. Consequently, regardless of whether the plant metric was measured as tree community composition or tree density, litter Ca content and litter cover were identified as significant predictors of ground-dwelling insect community composition. However, soil moisture content emerged as a significant predictor when plant metrics were based on tree community composition, but not when measured as tree density. This discrepancy is attributed to the high correlation between soil moisture content and understory tree density (Pearson’s *r* = 0.79).

### 3.4. The Diversity and Community Composition of Aerial Insects

Similar patterns in the family diversity and community composition of aerial insects were observed at the regional and local scales. At both scales, none of the measured diversity indices—including family richness index (^0^*D*), the exponential of Shannon’s index (^1^*D*), inverse Simpson’s index (^2^*D*), and inverse Berger–Parker index (^3^*D*)—showed significant differences between Masson pine plantations and natural forests (Figure 2B,D,F,H). No significant difference was observed in the community composition of aerial insects between Masson pine plantations and natural forests at both the regional (PERMANOVA: *F* = 0.85, *p* = 0.284; Figure 3B) and local (PERMANOVA: *F* = 1.50, *p* = 0.144; Figure 3D) scales.

## 4. Discussion

Our results showed that ground-dwelling and aerial insects exhibited distinct responses to differences in floristic composition and vegetation structure (and the associated litter properties) between Masson pine plantations and natural forests. Contrary to our hypothesis, there was no significant difference in the family diversity or composition of the aerial insect community between the two forest types. However, as hypothesized, a significant decline in family diversity of ground-dwelling insects and a notable shift in community composition were observed in Masson pine plantations compared to natural forests. These patterns were consistent at both regional and local scales. The local-scale experiment further revealed that the shift in community composition of ground-dwelling insects was primarily attributed to decreased understory tree density, reduced litter Ca content and increased litter cover in Masson pine plantations compared to natural forests. The distinct responses observed between ground-dwelling and aerial insects align with previous studies, which suggest that ground-dwelling insects are more sensitive to changes in vegetation than their aerial counterparts. For example, Akutsu et al. [35] revealed that logging disturbance (and the consequent vegetation changes) reduced the richness and abundance of understory insect groups, whereas no significant impact was detected on canopy insect communities. Similarly, Albacete et al. [36], in their study on the role of streams in non-riparian forest diversity conservation, demonstrated that vegetation structure at both tree and understory level was the dominant factor in shaping ground arthropod assemblages, whereas distance to the stream played a more significant role for aerial insect communities.

### 4.1. Ground-Dwelling Insects in Masson Pine Plantations

As hypothesized, the decline in family diversity of ground-dwelling insects in Masson pine plantations supports the well-documented pattern of reduced insect fauna in various pine plantations, which is attributed to their homogeneous vegetation structure and lower floristic diversity compared to natural forests [51,52,53]. For example, Paritsis and Aizen [51] revealed that the replacement of native *Nothofagus dombeyi* forests by exotic conifer plantations led to a decline in ground-dwelling beetle diversity in northwestern Patagonia, Chile. This decline was attributed to a more uniform tree size and a less diverse needle-leaf litter composition in conifer plantations. However, several studies have also found that ground-dwelling insect diversity in pine plantations is comparable to that in natural forests, which has been attributed to the environmental similarities between the two forest types [54,55,56,57]. For example, Martínez et al. [55] reported that pine plantations and natural oak forests in northern Spain’s Ibaizabal basin are structurally similar in their understory vegetation and litter layer, which leads to similar carabid assemblages. These seemingly contradictory findings may be reconciled by considering the ecosystem context in which plantations are established. For example, the latitude gradient in vegetation structure and floristic diversity may significantly mediate the ecological outcomes of plantations.

In this study, significant environmental differences were observed between Masson pine plantations and natural forests, with understory tree density, litter cover, and litter Ca content identified as key drivers of ground-dwelling insect assemblages. Of these, understory tree density was paramount, providing critical food resources (e.g., leaves, flowers, and fruits) and shelters for highly mobile ground-dwelling insects. The understory tree density in Masson pine plantations was only about half that of natural forests, which likely limited the availability of essential resources, thereby adversely affects these insect groups. For example, omnivores such as Drosophilidae and Nitidulidae, which have a detritivore-herbivore diet, utilize both live (e.g., tree sap) and dead (e.g., rotten fruits) plant substrates for food and refuge [36]. Their lower abundance in Masson pine plantations was likely a direct consequence of the scarce understory vegetation, which offered limited food and shelter. Our results are consistent with the findings of Albacete et al. [36], who confirmed that vegetation structure (particularly understory vegetation) is a key driver of ground arthropod communities. Specifically, they revealed that the abundance of ground omnivores and ground phytophages showed significant positive associations with tree density and understory cover, respectively. Furthermore, the reduced availability of herbivores and detritivores may trigger a trophic cascade, leading to declines in their predatory ground-dwelling insects such as Staphylinidae [36]. Notably, the difference in understory herbaceous plants between Masson pine plantations and natural forests could theoretically contribute to the difference in insect communities. However, this factor is considered negligible. The dense canopy in natural forests and a continuous mat of pine needles in plantations both strongly suppress herbaceous growth, resulting in less than 5% herbaceous cover in both forest types (personal observation) and thus minimal influence on insects.

Given that Ca is a major structural component crucial for insect development [58], the lower litter Ca content likely further contributed to the reduced ground-dwelling insect diversity in Masson pine plantations. Furthermore, although not directly measured, a similar reduction was likely occurred in live plant tissues (e.g., leaves, fruits). This comprehensive nutrient deficit may have impaired the development of both detritivores and herbivores, thereby suppressing the overall ground-dwelling insect diversity. Although no studies have specifically examined the impact of litter Ca on ground-dwelling insects, similar patterns have been observed for soil invertebrate communities. For example, elevated Ca content in leaf litter has been shown to increase earthworm densities [59], and variation in litter Ca content across forest types can shift the community composition of soil invertebrates in warm-temperate regions [58].

However, Masson pine plantations developed a continuous mat of decaying pine needles, exhibiting a higher litter cover (98.0%) than natural forests (91.0%). This abundant litter likely benefited crawling detritivores, as the litter layer offered them both a reliable food source in the form of decaying organic matter and critical refuge within the litter layer. Indeed, we observed a greater abundance of Blattidae and Gryllidae families, both of which are crawling detritivores [36], in Masson pine plantations. In line with our findings, Arnold et al. [60] demonstrated that cockroach richness and abundance increased with litter cover during the post-fire recovery of litter detritivores in *Eucalyptus* forests in southeastern Australia. In addition, Brouwers and Newton [61] revealed that the presence of crickets (Orthoptera: Gryllidae) was associated with a well-developed leaf litter layer and low ground vegetation cover on the Isle of Wight, UK. Nevertheless, the positive effect of high litter cover on crawling detritivores could not outweigh the negative effects on overall insect diversity arising from sparse understory vegetation and lower litter Ca content in Masson pine plantations.

### 4.2. Aerial Insects in Masson Pine Plantations

Although single-layered, even-aged monoculture plantations are also expected to disadvantage aerial insects, we found that family-level aerial insect communities in Masson pine plantations were comparable to those in natural forests. Our findings contrast with the reported decline in Lepidoptera diversity in *Pinus patula* plantations relative to natural forests in Ecuador [62], but align with observations of comparable mobile insect diversity in Scots pine plantations and semi-natural woodlands in the UK [63]. A potential mechanism for this lack of a disadvantageous effect is that highly mobile aerial insects can compensate for habitat simplicity by accessing resources across a mosaic of habitats, thereby reducing their reliance on specific forest types. In this study, many of the aerial insects captured by Malaise traps are highly mobile adults dedicated to reproduction. However, they spend the majority of their life cycle as immobile larvae within sheltered environments such as soils or plant galls. For example, Cicadellidae, Cecidomyiidae, and Sciaridae, the most abundant aerial insect taxa in both the two forest types, have sedentary larvae that depend on specific resources (e.g., galls, roots, soil fungi) [64,65]. However, their adults are highly mobile, with estimated dispersal distances of 100–250 m for Cicadellidae [66], 30–700 m for Cecidomyiidae [67], and 10–100 m for Sciaridae [68]. Given that the Masson pine plantations are located proximate to the natural forest in this study, the highly mobile adults can easily traverse the closely situated plantations and natural forests, allowing them to exploit both matrixes for resources and reproduction. As a result, aerial insects in Masson pine plantations may benefit from the proximity of natural forests through regular immigration and colonization, thereby they may exhibit weaker associations with specific forest types. Supporting this, Brandl et al. [69] found no significant differences in the abundance and species richness of cicadas among newly established grasslands with diverse seed mixtures, older grasslands, and subsidized legume-grass mixture grasslands in a study conducted in Austria to evaluate the effectiveness of agri-environmental schemes. These findings suggest that the buffering effect of surrounding vegetation is important for the maintenance of aerial insect assemblages in plantations.

However, the absence of a detectable decline in aerial insect diversity or a notable shift in community composition may be attributable to the coarse taxonomic resolution of this study. Family-level similarity likely masks considerable species-level diversity loss in Masson pine plantations. This is because, even within the same family, closely related species can exhibit markedly different microhabitat preferences and dietary specializations. The monospecific, single-layered structure of Masson pine plantations severely limits ecological niche availability, supporting only a narrow range of specialized niches. In contrast, natural forests, characterized by multi-layered vertical structure and diverse tree species, provide a much wider array of specialized niches. Given the strong host-plant specificity and sensitivity to vegetation structure typical of many phytophagous insects, Masson pine plantations likely support markedly different species assemblages (that nonetheless belong to the same families) and substantially lower overall species diversity than natural forests. For example, although Cicadellidae was a dominant family in both Masson pine plantations (mean relative abundance: 10.9%) and natural forests (mean relative abundance: 24.8%), the cicadellid species composition likely differed substantially between the two forest types. Masson pine plantations were probably dominated by needle-feeding specialists from genera such as *Grypotes*, *Shivapona*, and *Pinopona* [70]. In contrast, natural forests support a markedly different and more species-rich tree community, characterized by broadleaved taxa such as oaks, *Ficus* spp., and *Ampelopsis* spp., which are typically absent from Masson pine plantations. Consequently, natural forests are expected to host an entirely different assemblage of cicadellid species. For example, oaks likely support specialists from the genus *Aligia* [71], *Ficus* spp. likely harbor specialists from the genus *Ficocyba* [72], and *Ampelopsis* spp. may harbor species from the genus *Erythroneura* [73]. Therefore, accurate assessment of the biodiversity value of such plantations requires species-level identification and analysis in future studies.

## 5. Conclusions

A key caveat of this study is the reliance on family-level identification for assessing insect communities, an approach that likely underestimates true biodiversity loss in Masson pine plantations. While species- or genus-level resolution would certainly offer greater precision and ecological insight, the sheer volume of specimens rendered such fine-scale identification impractical for many groups, owing to intricate morphology, small body sizes, and limited availability of diagnostic keys or molecular resources—a well-documented challenge in insect taxonomy [74,75]. This limitation is not unique to our work; several previous studies assessing insect diversity in plantation systems have similarly relied on family-level metrics. For example, family richness has been used to evaluate the consequences of converting natural forests to rubber or *Eucalyptus* plantations for beetle communities in Laos [76], and to examine how management practices (e.g., thinning, mixed planting, alley-cropping) affect insect assemblages [77,78,79]. Nevertheless, family diversity remains ecologically meaningful in its own right, often effectively capturing functional diversity. Phylogenetic conservatism frequently generates functional redundancy within families, such that family-level metrics can detect disturbance responses as reliably as species-level data in many arthropod taxa (e.g., spiders, beetles, butterflies, moths, mites) [80,81]. In this context, although species-level metrics can offer deeper mechanistic understanding—an approach feasible perhaps only for selected taxa—family-level metrics provide a realistic, cost-effective, and methodologically robust alternative for assessing disturbance effects on insect communities and for informing ecosystem conservation and management. This approach aligns closely with Timms et al. [81], who emphasized in their synthesis of arthropod biodiversity research in temperate and boreal forests that higher-level taxonomy offers a practical trade-off for large-scale evaluations, particularly among poorly known groups, without sacrificing sensitivity to human-induced impacts.

In summary, family diversity and community composition of aerial insects in Masson pine plantations were comparable to those in natural forests. However, ground-dwelling insects exhibited a decline in family diversity and a notable shift in community composition, primarily due to reduced understory vegetation and nutrient-poor needle litter (particularly litter Ca). The lack of congruence in diversity patterns between ground-dwelling and aerial insects suggests that using a single indicator taxon to design or evaluate biodiversity conservation measures in plantations may be insufficient. Instead, conservation strategies should be tailored individually for different functional taxa. To enhance ground-dwelling insect diversity in Masson pine plantation, mixed planting with broad-leaved species represents an efficient management strategy. This approach both enriches litter nutrient content and reduces needle litter accumulation, thereby facilitating the recovery of understory vegetation. Given the taxonomic limitations of the present study, these findings should be further assessed at the species level to more accurately quantify biodiversity responses and evaluate the conservation value of Masson pine plantations.

## Figures and Tables

**Table 1:** Major insect taxa (contribution ≥ 2.0%) and their contributions to the dissimilarity in community composition of ground-dwelling insects between *Pinus massoniana* plantations and natural forests at the regional and local scales. Different letters in the same row indicate significant differences in the relative abundances between the two forest types (*p* < 0.05). The values are mean ± SE in the table.

Order	Family	Contribution (%)	Relative Abundance	
			*Pinus massoniana*	Natural Forests
Regional scale				
Blattodea	Blattellidae	23.6	0.387 ± 0.059 a	0.255 ± 0.048 b
Orthoptera	Gryllidae	18.9	0.293 ± 0.043 a	0.176 ± 0.043 b
	Tetrigidae	2.0	0.025 ± 0.014 a	0 b
Diptera	Drosophilidae	15.8	0.066 ± 0.031 b	0.192 ± 0.050 a
Coleoptera	Nitidulidae	7.9	0.038 ± 0.013 b	0.098 ± 0.027 a
	Scarabaeidae	4.9	0.014 ± 0.005 b	0.057 ± 0.023 a
	Staphylinidae	3.0	0.010 ± 0.003 b	0.037 ± 0.015 a
Local scale				
Diptera	Drosophilidae	27.4	0.018 ± 0.008 b	0.287 ± 0.083 a
	Lauxaniidae	3.5	0.013 ± 0.004 b	0.045 ± 0.014 a
Orthoptera	Gryllidae	16.7	0.279 ± 0.030 a	0.119 ± 0.036 b
Blattodea	Blattidae	13.2	0.264 ± 0.033 a	0.136 ± 0.030 b
	Polyphagidae	6.2	0.061 ± 0.006 a	0 b
	Blattellidae	5.0	0.007 ± 0.003 b	0.056 ± 0.012 a

**Table 2:** Summary of multiple regressions used to evaluate the significance of the selected explanatory variables after controlling for multicollinearity. The plant metric was measured as tree density.

Variable	Estimate	SE	*t*	*p*	Partial *R*^2^
Understory tree density	0.11	0.012	9.51	<0.001	0.459
Litter Ca	0.05	0.014	3.58	<0.001	0.297
Litter cover	0.07	0.011	5.88	<0.001	0.135

## Data Availability

The original contributions presented in this study are included in the article/Supplementary Material. Further inquiries can be directed to the corresponding author.

## Supplementary Materials

The following supporting information can be downloaded at: https://www.mdpi.com/article/10.3390/insects17020158/s1.

## References

[1] Mai C., Schmitt U., Niemz P. (2022). A brief overview on the development of wood research. Holzforschung.

[2] Besseau P., Graham S., Christophersen T. (2018). Restoring Forests and Landscapes: The Key to a Sustainable Future.

[3] Griscom B.W., Adams J., Ellis P.W., Houghton R.A., Lomax G., Miteva D.A., Schlesinger W.H., Shoch D., Siikamaki J.V., Smith P. (2017). Natural climate solutions. Proc. Natl. Acad. Sci. USA.

[4] Hua F., Wang X., Zheng X., Fisher B., Wang L., Zhu J., Tang Y., Yu D.W., Wilcove D.S. (2016). Opportunities for biodiversity gains under the world’s largest reforestation programme. Nat. Commun..

[5] Romijn E., Coppus R., De Sy V., Herold M., Roman-Cuesta R.M., Verchot L. (2019). Land restoration in Latin America and the Caribbean: An overview of recent, ongoing and planned restoration initiatives and their potential for climate change mitigation. Forests.

[6] Hua F., Bruijnzeel L.A., Meli P., Martin P.A., Zhang J., Nakagawa S., Miao X., Wang W., McEvoy C., Peña-Arancibia J.L. (2022). The biodiversity and ecosystem service contributions and trade-offs of forest restoration approaches. Science.

[7] Stephens S.S., Wagner M.R. (2007). Forest plantations and biodiversity: A fresh perspective. J. For..

[8] Brockerhoff E.G., Jactel H., Parrotta J.A., Quine C.P., Sayer J. (2008). Plantation forests and biodiversity: Oxymoron or opportunity?. Biodivers. Conserv..

[9] Bremer L.L., Farley K.A. (2010). Does plantation forestry restore biodiversity or create green deserts? A synthesis of the effects of land-use transitions on plant species richness. Biodivers. Conserv..

[10] Baker A.C., Murray B.R. (2012). Seasonal intrusion of litterfall from non-native pine plantations into surrounding native woodland: Implications for management of an invasive plantation species. For. Ecol. Manag..

[11] Brockerhoff E., Ecroyd C., Langer E. (2001). Biodiversity in New Zealand plantation forests: Policy trends, incentives, and the state of our knowledge. N. Z. J. For..

[12] Hartley M.J. (2002). Rationale and methods for conserving biodiversity in plantation forests. For. Ecol. Manag..

[13] Chu Van C., David L., Marc H. (2013). Simple plantations have the potential to enhance biodiversity in degraded areas of Tam Dao National Park, Vietnam. Nat. Areas J..

[14] Lopes I.T., Gussoni C.O.A., Demarchi L.O., de Almeida A., Pizo M.A. (2015). Diversity of understory birds in old stands of native and *Eucalyptus* plantations. Restor. Ecol..

[15] Cesar R.G., Moreno V.S., Coletta G.D., Chazdon R.L., Ferraz S.F.B., de Almeida D.R.A., Brancalion P.H.S. (2018). Early ecological outcomes of natural regeneration and tree plantations for restoring agricultural landscapes. Ecol. Appl..

[16] Pejchar L., Holl K.D., Lockwood J.L. (2005). Hawaiian honeycreeper home range size varies with habitat: Implications for native *Acacia koa* forestry. Ecol. Appl..

[17] Arrieta S., Suárez F. (2006). Scots pine (*Pinus sylvestris* L.) plantations contribute to the regeneration of holly (*Ilex aquifolium* L.) in mediterranean central Spain. Eur. J. For. Res..

[18] FAO (2020). Global Forest Resources Assessment 2020—Key Findings.

[19] Li Y., Dong Z., Chen D., Zhao S., Zhou F., Cao X., Fang K. (2019). Growth decline of *Pinus massoniana* in response to warming induced drought and increasing intrinsic water use efficiency in humid subtropical China. Dendrochronologia.

[20] Huang X., Huang C., Teng M., Zhou Z., Wang P. (2020). Net primary productivity of *Pinus massoniana* dependence on climate, soil and forest characteristics. Forests.

[21] Deng C., Zhang S., Lu Y., Froese R.E., Xu X., Zeng J., Ming A., Liu X., Xie Y., Li Q. (2020). Thinning effects on forest evolution in Masson pine (*Pinus massoniana* Lamb.) conversion from pure plantations into mixed forests. For. Ecol. Manag..

[22] Zhang Y., Li X., Zhang D., Qin Y., Zhou Y., Song S., Zhang J., Hui D. (2020). Characteristics of fungal community structure during the decomposition of mixed foliage litter from *Pinus massoniana* and broadleaved tree species in southwestern China. J. Plant Ecol..

[23] Ji L., Wang Z., Wang X., An L. (2011). Forest insect pest management and forest management in China: An overview. Environ. Manag..

[24] Chen L.-C., Liang M.-J., Wang S.-L. (2016). Carbon stock density in planted versus natural *Pinus massoniana* forests in sub-tropical China. Ann. For. Sci..

[25] Ming A., Yang Y., Liu S., Nong Y., Tao Y., Zeng J., An N., Niu C., Zhao Z., Jia H. (2020). A decade of close-to-nature transformation alters species composition and increases plant community diversity in two coniferous plantations. Front. Plant Sci..

[26] Wang Y., Zheng J., Liu X., Yan Q., Hu Y. (2020). Short-term impact of fire-deposited charcoal on soil microbial community abundance and composition in a subtropical plantation in China. Geoderma.

[27] Guo J., Gong X., Yu S., Wei B., Chu L., Liu J., He X., Yu M. (2023). Responses of soil microbial diversity to forest management practices after pine wilt disease infection. Forests.

[28] Keyhani A.B., He W., Teng M., Yan Z., Ma Z., Xu J., Fayaz M., Zhou C., Wei P., Wang P. (2024). Effect of mineral fertilizers on microorganisms community characteristic during leaf litter decomposition under *Pinus massoniana* in a subtropical forest. Appl. Soil Ecol..

[29] Dai D., Ali A., Huang X., Teng M., Wu C., Zhou Z., Liu Y. (2020). Soil available phosphorus loss caused by periodical understory management reduce understory plant diversity in a northern subtropical *Pinus massoniana* plantation chronosequence. Forests.

[30] Pan J., Guo Q., Li H., Luo S., Zhang Y., Yao S., Fan X., Sun X., Qi Y. (2021). Dynamics of soil nutrients, microbial community structure, enzymatic activity, and their relationships along a chronosequence of *Pinus massoniana* plantations. Forests.

[31] Stork N.E. (2018). How many species of insects and other terrestrial arthropods are there on Earth?. Annu. Rev. Entomol..

[32] Yang L.H., Gratton C. (2014). Insects as drivers of ecosystem processes. Curr. Opin. Insect Sci..

[33] A’Bear A.D., Jones T.H., Boddy L. (2014). Potential impacts of climate change on interactions among saprotrophic cord-forming fungal mycelia and grazing soil invertebrates. Fungal Ecol..

[34] Dray M.W., Crowther T.W., Thomas S.M., A’Bear A.D., Godbold D.L., Ormerod S.J., Hartley S.E., Jones T.H. (2014). Effects of elevated CO_2_ on litter chemistry and subsequent invertebrate detritivore feeding responses. PLoS ONE.

[35] Akutsu K., Khen C.V., Toda M.J. (2006). Assessment of higher insect taxa as bioindicators for different logging-disturbance regimes in lowland tropical rain forest in Sabah, Malaysia. Ecol. Res..

[36] Albacete S., Mac Nally R., Carles-Tolrá M., Domènech M., Vives E., Espadaler X., Pujadé-Villar J., Serra A., Maceda-Veiga A. (2020). Stream distance and vegetation structure are among the major factors affecting various groups of arthropods in non-riparian chestnut forests. For. Ecol. Manag..

[37] Yu X.D., Luo T.H., Yang J., Zhou H.Z. (2006). Distribution of ground-dwelling beetles (Coleoptera) across a forest-clearcut ecotone in Wolong Natural Reserve, southwestern China. Insect Sci..

[38] Cai B. (2017). Insect Taxonomy.

[39] Zheng L., Gui H. (1999). Insect Classification.

[40] Zhang W., Li Y. (2011). Chinese Insects Illustrated.

[41] Yang X. (2004). Insects from Mt. Shiwandashan Area of Guangxi.

[42] Wang Z., Qiu L., Che Y. (2024). Blattodea of China.

[43] Zahiri R., Holloway J.D., Kitching I.J., Lafontaine J.D., Mutanen M., Wahlberg N. (2011). Molecular phylogenetics of Erebidae (Lepidoptera, Noctuoidea). Syst. Entomol..

[44] Hill M.O. (1973). Diversity and evenness: A unifying notation and its consequences. Ecology.

[45] Chao A., Chiu C.-H., Jost L. (2014). Unifying species diversity, phylogenetic diversity, functional diversity, and related similarity and differentiation measures through Hill numbers. Annu. Rev. Ecol. Evol. Syst..

[46] Hsieh T.C., Ma K.H., Chao A., McInerny G. (2016). iNEXT: An R package for rarefaction and extrapolation of species diversity (Hill numbers). Methods Ecol. Evol..

[47] Bates D., Mächler M., Bolker B., Walker S. (2015). Fitting linear mixed-effects models using lme4. J. Stat. Softw..

[48] Oksanen J., Blanchet F.G., Kindt R., Legendre P., Minchin P.R., O’hara R., Simpson G.L., Solymos P., Stevens M.H.H., Wagner H. (2025). Vegan: Community Ecology Package. R Package Version 2.7.2. https://cran.r-project.org/web/packages/vegan/vegan.pdf.

[49] Xu W.B., Svenning J.C., Chen G.K., Zhang M.G., Huang J.H., Chen B., Ordonez A., Ma K.P. (2019). Human activities have opposing effects on distributions of narrow-ranged and widespread plant species in China. Proc. Natl. Acad. Sci. USA.

[50] Groemping U. (2006). Relative importance for linear regression in R: The package relaimpo. J. Stat. Softw..

[51] Paritsis J., Aizen M.A. (2008). Effects of exotic conifer plantations on the biodiversity of understory plants, epigeal beetles and birds in *Nothofagus dombeyi* forests. For. Ecol. Manag..

[52] Fierro A., Grez A.A., Vergara P.M., Ramírez-Hernández A., Micó E. (2017). How does the replacement of native forest by exotic forest plantations affect the diversity, abundance and trophic structure of saproxylic beetle assemblages?. For. Ecol. Manag..

[53] Cifuentes-Croquevielle C., Stanton D.E., Armesto J.J. (2020). Soil invertebrate diversity loss and functional changes in temperate forest soils replaced by exotic pine plantations. Sci. Rep..

[54] Fuller R.J., Oliver T.H., Leather S.R. (2008). Forest management effects on carabid beetle communities in coniferous and broadleaved forests: Implications for conservation. Insect Conserv. Diver..

[55] Martínez A., Iturrondobeitia J.C., Goldarazena A. (2009). Effects of some ecological variables on carabid communities in native and non native forests in the Ibaizabal basin (Basque Country: Spain). Ann. For. Sci..

[56] Giménez Gómez V.C., Verdú J.R., Gómez-Cifuentes A., Vaz-de-Mello F.Z., Zurita G.A. (2018). Influence of land use on the trophic niche overlap of dung beetles in the semideciduous Atlantic forest of Argentina. Insect Conserv. Diver..

[57] Swart R.C., Pryke J.S., Roets F. (2018). Arthropod assemblages deep in natural forests show different responses to surrounding land use. Biodivers. Conserv..

[58] Ohta T., Niwa S., Agetsuma N., Hiura T. (2014). Calcium concentration in leaf litter alters the community composition of soil invertebrates in warm-temperate forests. Pedobiologia.

[59] Reich P.B., Oleksyn J., Modrzynski J., Mrozinski P., Hobbie S.E., Eissenstat D.M., Chorover J., Chadwick O.A., Hale C.M., Tjoelker M.G. (2005). Linking litter calcium, earthworms and soil properties: A common garden test with 14 tree species. Ecol. Lett..

[60] Arnold K.T., Murphy N.P., Gibb H. (2016). Post-fire recovery of litter detritivores is limited by distance from burn edge. Austral Ecol..

[61] Brouwers N.C., Newton A.C. (2008). Habitat requirements for the conservation of wood cricket (*Nemobius sylvestris*) (Orthoptera: Gryllidae) on the Isle of Wight, UK. J. Insect Conserv..

[62] Adams M.-O., Fiedler K. (2015). The value of targeted reforestations for local insect diversity: A case study from the Ecuadorian Andes. Biodivers. Conserv..

[63] Humphrey J.W., Hawes C., Peace A.J., Ferris-Kaan R., Jukes M.R. (1999). Relationships between insect diversity and habitat characteristics in plantation forests. For. Ecol. Manag..

[64] Binns E.S. (1980). Field and laboratory observations on the substrates of the mushroom fungus gnat *Lycoriella auripila* (Diptera: Sciaridae). Ann. Appl. Biol..

[65] Skuhrava M., Skuhravy V. (2009). Species richness of gall midges (Diptera: Cecidomyiidae) in Europe (West Palaearctic): Biogeography and coevolution with host plants. Acta Soc. Zool. Bohem..

[66] Andrade S.C., Rossi G.D., Martinelli N.M. (2020). Dispersion pattern of giant cicada (Hemiptera: Cicadidae) in a Brazilian coffee plantation. Environ. Entomol..

[67] Hao Y.N., Miao J., Wu Y.Q., Gong Z.J., Jiang Y.L., Duan Y., Li T., Cheng W.N., Cui J.X. (2013). Flight performance of the orange wheat blossom midge (Diptera: Cecidomyiidae). J. Econ. Entomol..

[68] Hu J.R., Xie C., Shi C.H., Wang S.L., Wu Q.J., Li C.R., Zhang Y.J. (2019). Effect of sex and air temperature on the flight capacity of *Bradysia odoriphaga* (Diptera: Sciaridae). J. Econ. Entomol..

[69] Brandl M., Hussain R.I., Maas B., Rabl D., Pachinger B., Holzinger W., Krautzer B., Moser D., Frank T. (2022). Improving insect conservation values of agri-environment schemes through diversified seed mixtures. Biol. Conserv..

[70] Dietrich C.H., Dmitriev D.A. (2003). Reassessment of the leafhopper tribes Koebeliini Baker and Grypotini Haupt (Hemiptera: Cicadellidae). Ann. Entomol. Soc. Am..

[71] Bentz J.-A., Townsend A.M. (2005). Diversity and abundance of leafhopper species (Homoptera: Cicadellidae) among red maple clones. J. Insect Conserv..

[72] Koczor S., Schlitt B.P., Takács A., Kőszegi K., Medve Z., Kiss B. (2025). The fig leafhopper, *Ficocyba ficaria* (Horváth, 1897) established in Hungary (Hemiptera, Cicadellidae). Bull. Insectology.

[73] Vincent C., Lowery T., Parent J.-P. (2018). The entomology of vineyards in Canada. Can. Entomol..

[74] Jin M., Zwick A., Ślipiński A., de Keyzer R., Pang H. (2020). Museomics reveals extensive cryptic diversity of Australian prionine longhorn beetles with implications for their classification and conservation. Syst. Entomol..

[75] Sabatelli S., Ruspantini P., Cardoli P., Audisio P. (2021). Underestimated diversity: Cryptic species and phylogenetic relationships in the subgenus *Cobalius* (Coleoptera: Hydraenidae) from marine rockpools. Mol. Phylogenet. Evol..

[76] Chouangthavy B., Fourcade Y. (2023). Large-scale sampling of beetle communities in Laos shows that conversion of natural forests into plantations leads to a decline in family richness and abundance. Ecol. Evol..

[77] Maleque M.A., Ishii H.T., Maeto K., Taniguchi S. (2007). Line thinning fosters the abundance and diversity of understory Hymenoptera (Insecta) in Japanese cedar (*Cryptomeria japonica* D. Don) plantations. J. For. Res..

[78] Damptey F.G., Opuni-Frimpong E., Nsor C.A., Addai J., Debrah D.K., Schnerch B., Bentsi-Enchill F., Korjus H. (2022). Taxonomic and community composition of epigeal arthropods in monoculture and mixed tree species plantations in a deciduous forest of Ghana. J. For. Res..

[79] Ashraf M., Zulkifli R., Sanusi R., Tohiran K.A., Terhem R., Moslim R., Norhisham A.R., Ashton-Butt A., Azhar B. (2018). Alley-cropping system can boost arthropod biodiversity and ecosystem functions in oil palm plantations. Agric. Ecosyst. Environ..

[80] Caruso T., Migliorini M. (2006). Micro-arthropod communities under human disturbance: Is taxonomic aggregation a valuable tool for detecting multivariate change? Evidence from Mediterranean soil oribatid coenoses. Acta Oecol..

[81] Timms L.L., Bowden J.J., Summerville K.S., Buddle C.M. (2013). Does species-level resolution matter? Taxonomic sufficiency in terrestrial arthropod biodiversity studies. Insect Conserv. Diver..

