# Supplementary material for: Contrasting Conservation Outcomes for Ground-Dwelling and Aerial Insects in Masson Pine Plantations: Reduced Ground-Dwelling Insect Diversity but Comparable Aerial Insect Diversity to Natural Forests
Source: Insects. 2026 Feb 2;17(2):158. doi: 10.3390/insects17020158 (PMC12941260; doi:10.3390/insects17020158)
Supplement: Supplementary file 1 [file insects-17-00158-s001.zip › insects-4081629-supplementary.pdf]

Supplementary Information for

Contrasting conservation outcomes for ground-dwelling and aerial insects in Masson pine plantations: reduced ground-dwelling insect diversity but comparable aerial insect diversity to natural forests

This word file includes:

Figure S1 to S3

Table S1 to S4

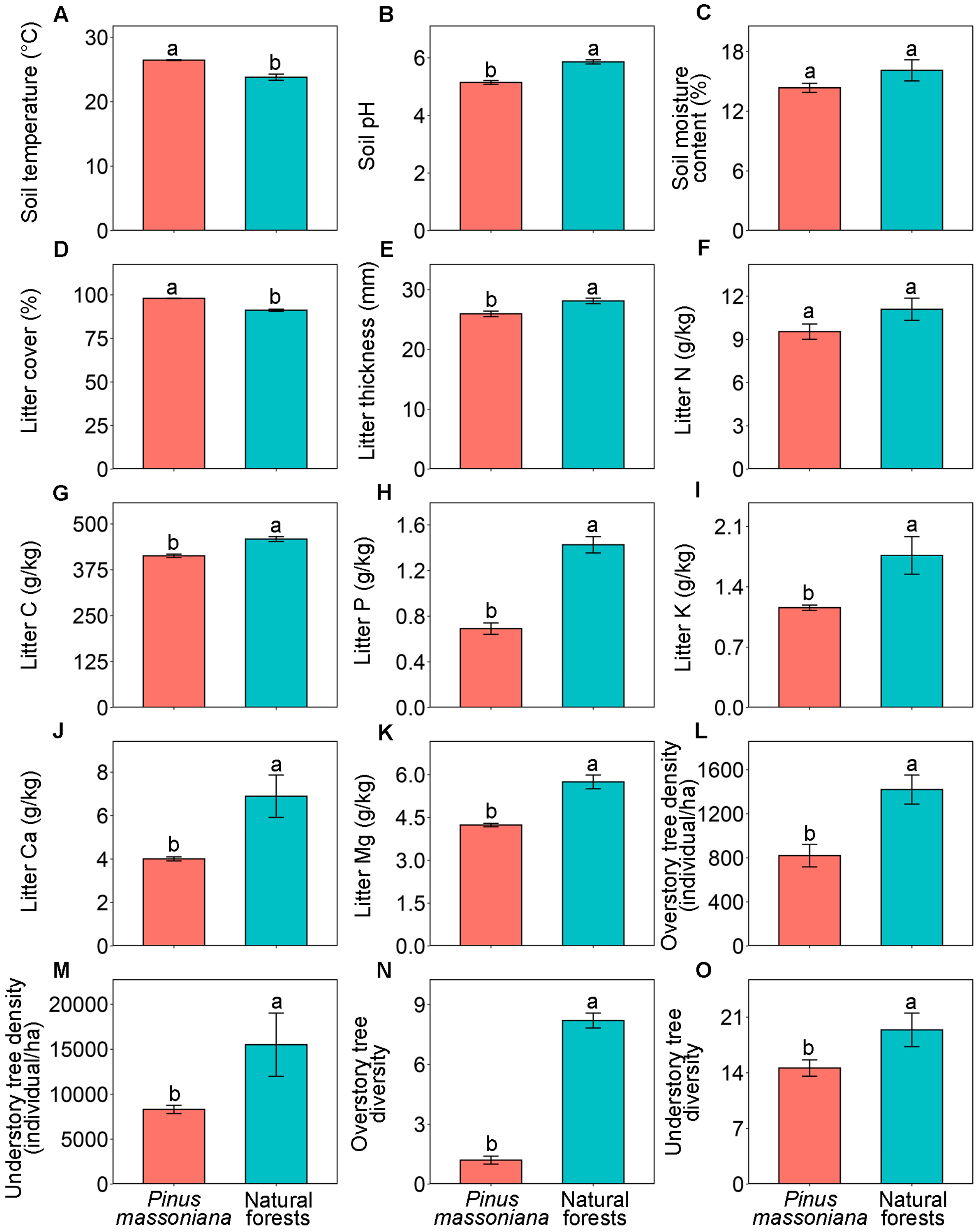

Figure S1 Soil properties, litter properties and vegetation structure of *Pinus massoniana* plantations and natural forests at the local scale at Yachang, Guangxi, China. Different letters indicate significant differences between the two forest types at $P < 0.05$.

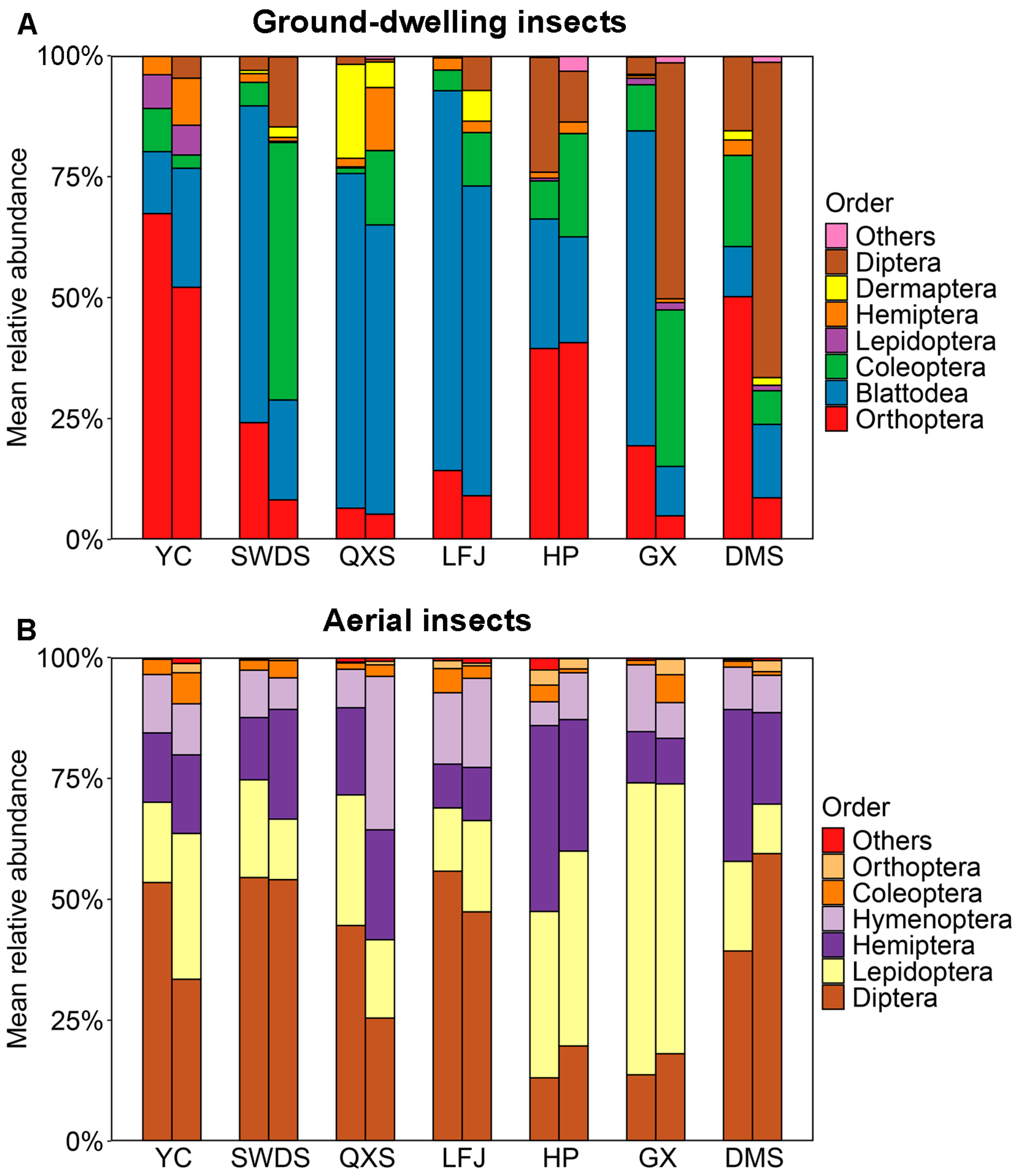

Figure S2 The mean relative abundance of dominant ground-dwelling (A) and aerial (B) insect taxa at the order level in *Pinus massoniana* plantations (left bars) and natural forests (right bars) at the regional scale across Guangxi, China. Only those orders with a mean relative abundance >1.0% across all samples are shown, while all remaining orders are grouped as others. SWDS: Shiwandashan Nature Reserve, DMS: Damingshan Nature Reserve, HP: Huaping Nature Reserve, GX: Guxiu Nature Reserve, YC: Yachang Orchid Nature Reserve, QXS: Qingxiushan Forest Park, LFJ: Liangfengjiang Forest Park.

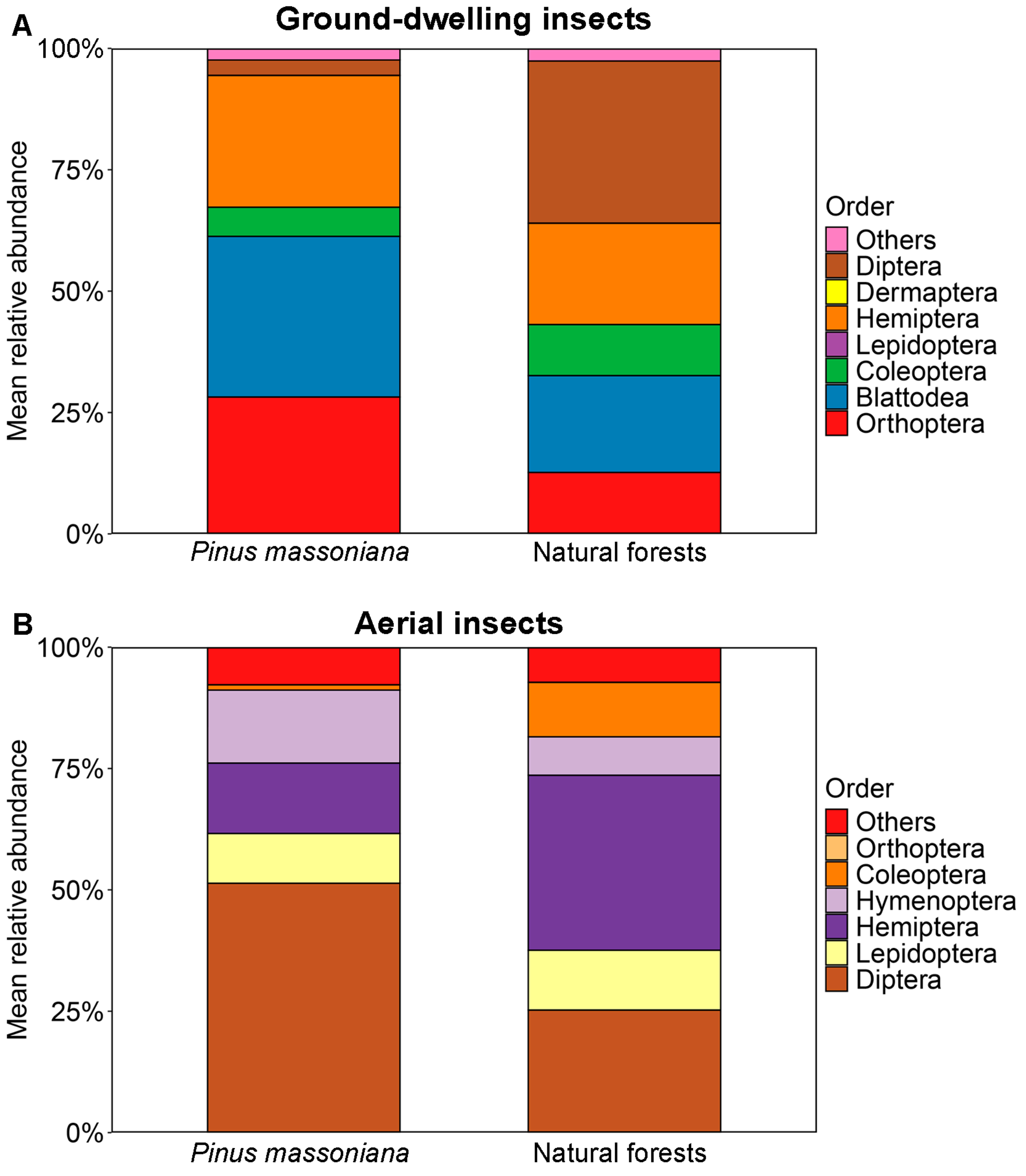

Figure S3 The mean relative abundance of dominant ground-dwelling (A) and aerial (B) insect taxa at the order level in *Pinus massoniana* plantations and natural forests at the local scale at Yachang, Guangxi, China. Only those orders with a mean relative abundance >1.0% across all samples are shown, while all remaining orders are grouped as others.

Table S1 Location, climate and vegetation types of the study sites in Guangxi, China. SWDS: Shiwandashan Nature Reserve, DMS: Damingshan Nature Reserve, HP: Huaping Nature Reserve, GX: Guxiu Nature Reserve, YC: Yachang Orchid Nature Reserve, QXS: Qingxiushan Forest Park, LFJ: Liangfengjiang Forest Park.

Site	Longitude	Latitude	Mean annual temperature (°C)	Mean annual precipitation (mm)	Vegetation type
SWDS	107°53'24"E	21°50'45"N	21.4	2488.1	Northern tropical seasonal rainforest
DMS	108°25'48"E	23°29'55"N	17.2	2221.4	Southern subtropical evergreen broad-leaved forest
HP	109°54'13"E	25°37'5" N	16.5	1766.7	Northern subtropical evergreen broad-leaved forest
GX	110°31'7"E	24°14'7"N	21.4	1925.5	Mid-subtropical evergreen broad-leaved forest
YC	106°19'36"E	24°50'32"N	18.4	1390.3	Mid-subtropical mixed evergreen and deciduous broad-leaved forest
QXS	108°23'24"E	22°46'52"N	23.1	1849.5	Southern subtropical evergreen broad-leaved forest
LFJ	108°16'46"E	22°43'19"N	23.2	1910.0	Southern subtropical evergreen broad-leaved forest

Table S2 Summary of the linear regressions between ground-dwelling insect community dissimilarity and environmental dissimilarities (plant, soil, and litter properties). The ground-dwelling insect community dissimilarity was measured by Bray–Curtis distances, while environmental dissimilarities were measured by Euclidean distances. $P < 0.05$ are shown in bold.

Variable	R^2	P
Soil temperature	0.117	**0.021**
Soil moisture content	0.523	**< 0.001**
Soil pH	0.232	**< 0.001**
Litter cover	0.215	**0.001**
Litter thickness	0.003	0.715
Litter C	0.041	0.180
Litter N	0.003	0.723
Litter P	0.438	**< 0.001**
Litter K	0.056	0.119
Litter Ca	0.596	**< 0.001**
Litter Mg	0.143	**0.010**
Understory tree community	0.478	**< 0.001**
Overstory tree community	0.445	**< 0.001**
Understory tree density	0.716	**< 0.001**
Overstory tree density	0.003	0.737

Table S3 Summary of the pairwise Pearson's correlation analysis for detecting the collinearity between pairs of independent variables (i.e., environmental dissimilarities). The understory and overstory tree community dissimilarities were measured by Bray–Curtis distances, while other environmental dissimilarities were measured by Euclidean distances. Pearson's $r \geq 0.7$ are shown in bold. SMC: Soil moisture content, UTC: Understory tree community, OTC: Overstory tree community, UTD: Understory tree density, OTD: Overstory tree density.

Variable	Soil temperature	SMC	Soil pH	Litter cover	Litter thickness	Litter C	Litter N	Litter P	Litter K	Litter Ca	Litter Mg	UTC	OTC	UTD	OTD
Soil temperature	1														
SMC	-0.01	1													
Soil pH	**0.81**	0.03	1												
Litter cover	0.69	0.11	**0.75**	1											
Litter thickness	0.24	0.08	0.22	0.63	1										
Litter C	0.42	-0.06	0.47	**0.81**	0.69	1									
Litter N	0.04	-0.04	0.14	0.04	-0.20	0.12	1								
Litter P	0.58	0.25	**0.74**	**0.83**	0.33	0.59	0.26	1							
Litter K	**0.81**	0.08	0.46	0.31	0.05	0.05	-0.10	0.19	1						
Litter Ca	0.36	0.68	0.43	0.30	-0.04	0.01	0.09	0.53	0.34	1					
Litter Mg	**0.98**	0.01	**0.86**	**0.72**	0.26	0.44	0.04	0.62	**0.78**	0.36	1				
UTC	0.47	0.48	0.49	0.63	0.24	0.49	0.14	0.66	0.21	0.67	0.47	1			
OTC	**0.75**	0.25	**0.79**	**0.91**	0.40	**0.73**	0.16	**0.85**	0.42	0.50	**0.77**	**0.78**	1		
UTD	0.00	**0.79**	0.09	0.10	-0.14	-0.10	0.09	0.39	0.06	0.65	0.04	0.53	0.33	1	
OTD	0.23	-0.10	0.18	0.62	0.44	0.65	-0.04	0.30	-0.02	-0.08	0.18	0.37	0.47	-0.15	1

Table S4 Summary of multiple regressions used to evaluate the significance of the selected explanatory variables after controlling for multicollinearity. The plant metric was measured as tree community composition. *P* values < 0.05 are shown in bold.

Variable	Estimate	SE	*t*	*P*	Partial R^2
**Initial model:**	**R^2 = 0.753, *P* < 0.001**				
Litter Ca	0.07	0.024	2.78	**0.008**	0.250
Soil moisture content	0.09	0.024	3.72	**< 0.001**	0.245
Understory tree community	0.19	0.232	0.82	0.415	0.168
Litter cover	0.05	0.022	2.31	**0.026**	0.090
**Final model:**	**R^2 = 0.749, *P* < 0.001**				
Litter Ca	0.08	0.022	3.55	**< 0.001**	0.322
Soil moisture content	0.09	0.024	3.94	**< 0.001**	0.294
Litter cover	0.06	0.017	3.58	**< 0.001**	0.133
